# *NEXN* Is a Novel Susceptibility Gene for Coronary Artery Disease in Han Chinese

**DOI:** 10.1371/journal.pone.0082135

**Published:** 2013-12-11

**Authors:** Chong Wu, Han Yan, Jingzhi Sun, Fan Yang, Chun Song, Feng Jiang, Yang Li, Jie Dong, Gu-Yan Zheng, Xiao-Li Tian, Huiqing Cao

**Affiliations:** 1 Department of Human Population Genetics, Institute of Molecular Medicine, Peking University, Beijing, China; 2 Department of Cardiolody, Affiliated Hospital of Jining Medical University, Jining, China; 3 Department of Cardiology, Chaoyang Hospital, Capital Medical University, Beijing, China; William Harvey Research Institute, Barts and The London School of Medicine and Dentistry, Queen Mary University of London, United Kingdom

## Abstract

Coronary artery disease (CAD) is the leading cause of death and disability in the world. Genome-wide association studies have implicated the importance of the genetic contribution of vascular smooth muscle cells (VSMCs) function in CAD susceptibility. The aberrant phenotypic modulation of VSMC is responsible for the pathological vascular intima hyperplasia that is the hallmark for atherosclerotic morphology. NEXN is a muscle-specific F-actin binding protein and is regulated by inflammatory cytokines in VSMCs. Whether NEXN contributes to human vascular disorders is still unknown. In this study, we genotyped 5 SNPs, tagging all of the 17 common SNPs within 54 kilobases (kb) covering *NEXN* gene and its flanking region, in 1883 patients with CAD and 1973 healthy individuals from Han Chinese, and identified one SNP, rs1780050, which was strongly associated with CAD trait. The Bonferroni corrected P-value was 7.65×10^−5^. The odds ratio (95% confidence interval) was 1.23 (1.12–1.36) with statistical power of 0.994. Functional analysis showed that NEXN promotes VSMC to a contractile phenotype *in vitro* and inhibits balloon-injury induced neointima formation *in vivo*. Further eQTL analysis demonstrated that the risk allele T of rs1780050 is associated with decreased expression of NEXN, thus contributing to a higher risk of CAD susceptibility in the population. This is, to our knowledge, the first study to identify *NEXN* as a novel CAD susceptibility gene with both genetic and functional evidence.

## Introduction

Coronary artery disease (CAD) is the leading cause of death in the world, making a worldwide health concern [1]. As a complex disease, both genetic and environmental factors contribute to CAD susceptibility. It is estimated that heritable factors account for 30%–60% of the inter-individual variation in the risk of CAD [2]. Searching for the genetic determinants has been considered an important step for the understanding of CAD. Over the past years, both genome-wide and candidate-gene-based association studies have successfully identified a number of novel chromosome loci or genes associated with CAD [3], [4], but they account for a relatively small portion of the overall CAD risk, novel loci or genes remain to be identified.

Vascular smooth muscle cells (VSMCs) are the major constituents participating in the atherosclerotic process. VSMCs exist in different phenotypes. The phenotypic switch of SMCs from a quiescent, “contractile” state to a proliferative “synthetic” state has been shown to play a key role in the vascular repair, pathogenesis of atherosclerosis and plaque rupture [5]. Recent genetic findings demonstrated the importance of VSMC function in CAD susceptibility. 9p21 is the most consistently replicated genetic locus for CAD. VSMCs that are deleted with 9p21 exhibit excessive proliferation, indicating a pivotal role of the locus in maintaining the differentiation phenotype of VSMCs [6]. ADAMTS7, which functions as a disintegrin and metalloproteinase, is identified as a novel CAD candidate by independent GWAS studies [7]–[9]. A variant of ADAMTS7 inhibits VSMC migration and is associated with CAD protection [10]. Thus we presumed that genes involved in VSMC phenotypic modulation are potential candidates for coronary artery disease.

NEXN is an F-actin binding protein localized at cell-matrix adherens junction. We previously reported that it is highly expressed in muscle [11]. The role of NEXN in heart has been well established in Z-disc stabilization and force generation, and mutant NEXN in patients leads to cardiomyopathies [12], [13]. There is evidence suggesting that NEXN is functional in pathological process of VSMCs [14], [15]. However, it is unknown whether or not *NEXN* is associated with susceptibility of coronary artery disease.

In the present study, we for the first time identified NEXN as a novel CAD susceptibility gene in Han Chinese population using both genetic and functional approaches, which will enhance our understanding of the etiology of CAD in humans.

## Materials and Methods

### Ethics Statement

Signed consent form was obtained from each participant. The study protocols were approved by the local hospital ethics committees: Ethics Committee of Chaoyang Hospital, Capital Medical University; Ethics Committee of The First Affiliated Hospital, China Medical University and Ethics Committee of The Fourth Affiliated Hospital of Harbin Medical University. The study conformed to the principles outlined in the Declaration of Helsinki.

All animal experiment procedures in this study were approved by the Institutional Animal Care and Use Committee (IACUC) of Peking University accredited by AAALAC International (IACUC No.: IMM-Tian XL-04).

### Populations and the clinical assessment of risk factors

All of the 1883 CAD patients in this study were hospitalized patients from three medical centers in north-eastern and northern China, Harbin, Shenyang and Beijing, respectively. Clinical definition of CAD and risk factors has been previously described in detail [16]. Briefly, coronary artery disease was defined according to one of the following criteria: existing myocardial infarction; treated with PCI (percutaneous coronary intervention) or CABG (coronary artery bypass graft); more than 50% diameter stenosis of at least one of the three major coronary arteries demonstrated angiographically. The 1973 controls were selected from the cohorts with at least five-year follow-up of general physical exam in three medical centers if the following criteria were simultaneously met: no family history for CAD; no clinical symptoms (angina, chest pressure, or shortness of breath) for CAD; and normal morphology of the resting electrocardiogram (EKG). Those with stroke, peripheral vascular diseases, and kidney diseases were excluded in this study.

Blood pressure was measured by the standard mercury sphygmomanometer, and carried out for 3 times at an interval of 5 minutes between each. The mean of three reads was calculated as the final values of blood pressures (systolic and diastolic blood pressure). Hypertension was defined as under antihypertensive treatment or SBP and DBP were equal to or greater than140 and/or 90 mmHg confirmed by three tests at different days. Diabetes mellitus was diagnosed according to the criteria of the American Diabetes Association (taking hypoglycemic agents, or fasting serum glucose level ≥7.0 mmol/L or a 2-hour postprandial glucose level ≥11.1 mmol/L in two measurements). Those with fasting serum glucose level in the range of 5.6–7.0 mmol/L or a 2-hour postprandial glucose level in 7.8–11.1 mmol/L were subjected to oral glucose tolerance test for inclusion or exclusion. Hyperlipidemia was defined as receiving treatment or low density lipoprotein cholesterol ≥130 mg/dL or total cholesterol ≥200 mg/dL. Smoking was grouped by never smoking and smoking based upon self-reports. Body mass index (BMI) was measured according to the formula: body weight (kg)/height^2^ (m^2^).

### SNP Selection and Genotyping

Human genomic DNA was isolated from blood using the proteinase K methods described previously [17]. Based on the HapMap (CHB), the 5 SNPs spanning 54 kb covering the gene region of NEXN and its flanking region were selected, meeting following criteria: (1) tagging SNPs based on r^2^≥0.8; (2) functional if possible; and (3) minor allele frequency (MAF)≥10%.

Two genotyping methods, direct sequencing and MLPA were used to avoid the bias caused by single genotyping method. 10 ng of genomic DNA from each participant was used to amplify DNA fragments of 150–250 bp containing the SNPs by PCR with the primers listed (Table S1). After purification by PEG precipitation, the DNA fragment was subjected to direct sequencing on ABI 3130XL according to the manual description of BigDye v3.1 kit. MLPA was according to the description by Schouten [18]. PCR primers and probes used for MLPA were listed in Table S2. Sequences of fluorescent universal primers were 5′-FAM-GGATA CGACT CACTA TAGGT -3′ and 5′-Hex-GCGGA TAACA AGTTC ACACT-3′.

### Cell Culture and Treatment

VSMCs were isolated from the thoracic aortas of 120 g to 150 g male Sprague-Dawley rats by enzymatic dissociation as described previously [19]. Cells were grown in DMEM supplemented with 10% (vol/vol) heat-inactivated FBS, 100 U/ml penicillin, and 100 µg/ml streptomycin. Cultures were maintained at 37°C in a humidified 95% air-5% CO2 atmosphere. VSMS between passages 3–5 were used for experiments. Cells were quiesced by incubating in DMEM containing 0.1% calf serum for 72 hours and used to perform the experiments with growth factors. PDGF-BB, TGF-β and FGF2 were purchased from Peprotech Inc (Rocky Hill, NJ). For adenovirus treatment, VSMCs were quiesced and transduced with Adv-Nexn and Adv-GFP control at a m.o.i of 50. Experiments were performed in triplicate.

### Adenoviral Constructs

The full-length Nexilin was cloned by PCR from the cDNA of rat VSMCs and inserted into pShuttle-IRES-hrGFP-1 with a flag tag in the C-terminus. Adenovirus was generated in an Ad-easy system (Stratagene) according to the manufacturer's protocol. PCR primers for rat Nexn clone were listed in Table S3.

### Reverse-transcriptase PCR

Total RNA was isolated from cells using TRIzol reagent (Invitrogen). 5 µg of RNA was digested with RNase-free DNase I and then reverse transcribed to cDNA using random primers and M-MLV reverse transcriptase (TransGen). For semi quantitative RT-PCR analysis, target genes were amplified using the primers listed in Table S4. 18S rRNA was used for normalization. The linear ranges of cycle number for target genes and 18 s were determined by preliminary tests. The PCR program was: 95°C for 3 min, 95°C for 15 sec, 60°C for 30 sec, 72°C for 30 sec, 20 cycles for 18 s and 27–35 cycles for target genes followed by dissociation. The intensities of the ethidium-bromide-stained bands were quantified using Gel-Pro Analyzer. Each experiment was performed in triplicate and a typical result was shown.

### Immunoblotting

Immunoblotting was performed by a standard method. Total protein lysates were prepared from cells or tissues with protease inhibitors followed by sonication. Equal amounts of protein (30 ug) were loaded onto a SDS-PAGE, transferred to PVDF membrane and subsequently blocked in 5% BSA. Primary antibodies to NEXN (BD Biosciences, CA, USA) and GAPDH (Santa Cruz, USA) were diluted in 5% BSA, followed by the secondary antibodies IRDye 680CW-conjugated goat anti-mouse IgG or IRDye 800CW-conjugated goat anti-rabbit IgG (LI-COR, USA). The blots were visualized using Odyssey (LI-COR, USA).

### MTT Assay

RASMC were plated and infected with Adv-NEXN or Adv-GFP for 12 hours, followed by serum starvation for another 48 hours. MTT assay was performed to obtain the cell viability index as described [20].

### Rat Carotid Artery Balloon Injury

Balloon injury was performed as described previously [21]. After balloon injury, solutions of (100 µL) Ad-GFP and Ad-NEXN (1010 pfu/mL) were infused into the ligated segment of the common carotid artery for 30 minutes. The ligatures and catheter were then removed. For morphometric analysis, carotid arteries were fixed in 10% formalin and embedded in paraffin. Sections (5-µm thick) obtained at equally spaced intervals in the middle of injured and control common carotid artery segments were stained with hematoxylin/eosin. The intimal and medial areas were measured using image J software and the intimal/medial ratios were calculated.

### F-actin staining

Cells were fixed with 4% paraformaldehyde and permeabilized in 0.3% Triton X-100, F-actin was stained with phalloidin-tetramethylrhodamine B (Sigma, USA). Fluorescence images were acquired on a Zeiss (Jena, Germany) LSM 700 confocal microscope with a ×40 objective at an axial resolution of 1.0 µm.

### eQTL analysis

We randomly recruited 121 healthy individuals from the populations described above. DNA was extracted and rs1780050 was genotyped by direct sequencing. Total RNA was isolated from blood lymphocytes and purified according to the TRIzol reagent method described above. The amount of NEXN transcripts were quantified by real-time PCR relative to 18S and further normalized by a standard method. The relative expression of NEXN was presented as normalized Ct, which was inversely correlated with the true amount of NEXN transcripts.

### Statistical Analysis

Hardy–Weinberg equilibrium test for all SNPs was calculated using the Chi-square test. Differences in allele and genotype distribution between CAD patients and controls were analyzed using binary logistic regression under various genetic models. Multiple comparison correction was calculated using Bonferroni correction. The two-tailed P values, odds ratios, and 95% confidence intervals (95% CI) are presented for all association tests.

Results of functional study were expressed as mean ± SD on the basis of at least triplicate experiments. Statistical analysis was made using Student's t-test (2-tailed). A value of P<0.05 was considered statistically significant.

## Results

### Characteristics of populations

A total of 1883 CAD patients and 1973 controls of Han Chinese were recruited from three medical centers in north-eastern and northern China. We designed a 2-stage genetic association study, a discovery stage and a following replication stage, to test whether *NEXN* is associated with CAD. Population 1 of the discovery stage consisted of 450 CAD cases and 450 gender- and age-matched controls from north-eastern China (Harbin City). Population 2 and 3 of the replication stage comprised 656 CAD cases versus 740 controls, and 777 cases versus 783 controls from north-eastern (Shenyang City) and northern China (Beijing City), respectively. Student's t-test was used to compare between cases and controls for age and BMI, and Chi-square test was calculated for comparison of hypertension, diabetes, hyperlipidemia and smoking. A mean ± SEM for age and BMI as well as percentages for other risk factors were presented in Table 1.

**Table 1:** Characteristics of populations in this study.

	Population 1			Population 2			Population 3		
	case	control	P	case	control	P	case	control	P
	(n = 450)	(n = 450)		(n = 656)	(n = 740)		(n = 777)	(n = 783)	
**Male(%)**	307(68.2%)	309(68.7%)	0.840	511(77.9%)	557(75.3%)	0.248	553(71.1%)	511(65.2%)	0.012
**Age(Year)**	55.8±0.4	57.2±0.5	0.050	60.5±0.4	62.1±0.4	0.009	53.6±0.3	53.9±0.3	0.497
**BMI(kg/m^2^)**	26.3±0.2	25.7±0.2	0.005	25.6±0.1	24.9±0.1	<0.001	25.6±0.1	24.3±0.1	<0.001
**HT(%)**	293(65.1%)	186(41.3%)	<0.001	424(64.6%)	321(43.4%)	<0.001	486(62.5%)	321(25.9%)	<0.001
**DM(%)**	172(38.2%)	139(30.9%)	0.021	218(33.2%)	120(16.2%)	<0.001	192(24.7%)	51(6.5%)	<0.001
**HLP(%)**	235(52.2%)	173(38.4%)	<0.001	276(42.1%)	358(48.4%)	0.018	489(62.9%)	387(49.4%)	<0.001
**Smoking(%)**	249(55.3%)	213(47.3%)	<0.001	351(53.5%)	336(45.4%)	<0.001	371(47.7%)	266(34.0%)	<0.001

± SEM (standard error of the mean). P values for age and BMI are calculated using Student's t-test. P values for hypertension, diabetes, hyperlipidemia and smoking are calculated by Chi-square test. BMI: body mass index; HT: hypertension; DM: diabetes mellitus; HLP: hyperlipidemia. The data for age and BMI are presented as mean

### Single SNP association test

We first selected and genotyped 5 SNPs in 54 kb region covering NEXN gene and its flanking sequence by sequencing in 450 CAD cases versus 450 controls of population 1, from north-eastern China. All SNPs passed the Hardy–Weinberg equilibrium test (Table 2). In allelic association analysis, two of the five SNPs, rs1166706 and rs1780050, presented the positive signals for association with CAD in regression test (with adjustment for other risk factors including age, BMI, hypertension, diabetes, hyperlipidemia and smoking) (Table 2). In addition, genotyping results by direct sequencing were also validated by MLPA with consistency >99.9%, avoiding the possible bias caused by a single genotyping method.

**Table 2:** Allelic association of 5 SNPs in Population 1 of discovery stage.

SNP	Position	Allele (major/minor)	MAF (case/control)	HWE P	P	OR (95% CI)
rs1166706	78131617	C/T	0.104/0.137	0.574	0.036	0.72(0.53–0.98)
rs1780045	78146989	C/T	0.097/0.083	0.589	0.514	1.12(0.80–1.58)
rs1166698	78165034	A/G	0.467/0.447	0.734	0.380	1.09(0.90–1.33)
rs1780050	78173128	G/T	0.511/0.459	0.169	0.016	1.27(1.04–1.55)
rs17101082	78173795	T/C	0.107/0.114	0.678	0.600	0.92(0.67–1.26)

% CI): odds ratio with 95% confidence interval. SNP position was based on NCBI B36 assembly, dbSNP b126. MAF: minor allele frequency; HWE P: P value from Chi-square for Hardy-Weinberg Equilibrium test; P: P value from logistic regression after adjustment for gender, age, BMI, hypertension, diabetes, hyperlipidemia and smoking; OR (95

### Replication study and genotypic association test

To validate the association, we selected 656 CAD cases versus 740 controls, and 777 cases versus 783 controls from north-eastern (Shenyang City) and northern China (Beijing City) for population 2 and 3 (Table 1). One of the two SNPs, rs1780050, showed the positive signals for association with CAD in both population 2 and 3. However, rs1166706 failed to be validated in population 2 and 3 of replication stage, suggesting a false positive signal in the discovery stage for this SNP. In a combined analysis, the Bonferroni-corrected P value for rs1780050 was 7.65×10^−5^, the statistical power was 0.994 (Table 3). Genotypic association analysis was carried out to test the possible genetic models by which rs1780050 may act (Table 4). Interestingly, rs1780050 presented association with CAD in all of the three models; however, the association was more significant in both dominant and additive genetic models. Taken together, we validated that one of the two positive SNPs from discovery stage, rs1780050, is associated with CAD in Han Chinese population and the minor allele might act by dominant or additive models.

**Table 3:** Allelic association of rs1166706 and rs1780050 with CAD in Population 2 and 3 of replication stage.

SNP	Population 2				Population 3				Three Populations Combined			
	MAF (case/control)	HWE P	P	OR (95% CI)	MAF (case/control)	HWE P	P	OR (95% CI)	MAF (case/control)	P/power	OR (95% CI)	bP
rs1166706	0.097/0.101	0.839	0.937	0.99(0.76–1.29)	0.115/0.113	0.475	0.930	0.99(0.77–1.27)	0.106/0.114	0.247/N.D	0.91(0.79–1.06)	N.D
rs1780050	0.502/0.449	0.405	0.008	1.24(1.06–1.45)	0.515/0.469	0.132	0.034	1.18(1.01–1.39)	0.510/0.459	1.53×10–5/0.994	1.23(1.12–1.36)	7.65×10−5

% CI): odds ratio with 95% confidence interval; power: statistic power when alpha is set as 0.05; bP: corrected P by Bonferroni method; N.D: not determined. MAF: minor allele frequency; HWE P: P value from Chi-square for Hardy-Weinberg Equilibrium test; P: P value from logistic regression after adjustment for gender, age, BMI, hypertension, diabetes, hyperlipidemia and smoking; OR (95

**Table 4:** Genotypic association analysis of rs1166706 and rs1780050 with CAD in three populations combined.

	Model	P	OR(95% CI)
rs1166706	Dom	0.424	0.94(0.79–1.10)
	Rec	0.052	0.48(0.23–1.01)
	Add	0.239	0.91(0.78–1.06)
rs1780050	Dom	1.81×10^−5^	1.40(1.20–1.63)
	Rec	0.006	1.25(1.07–1.47)
	Add	1.37×10^−5^	1.24(1.12–1.36)

% CI): odds ratio with 95% confidence interval. Dom: dominant model; Rec: recessive model; Add: additive model; P: P-value from logistic regression, adjusted by gender, age, BMI, hypertension, diabetes, hyperlipidemia and smoking; OR (95

### Assessment of CAD risk factors

In order to understand the interaction between genetic variants and other risk factors, we carried out subgroup analysis on SNP rs11780050. We found that rs1780050 was significantly associated with non-hypertensive group with a P value of 1.49×10^−5^, while in the hypertensive group the P value is above 0.05. The other risk factors, such as gender, age, obesity, diabetes, hyperlipidemia and smoking, seemed not to enhance the susceptibility for CAD in the individuals who carried rs1780050 (Table 5).

**Table 5:** Subgroup analysis of rs1780050 in combined populations.

Subgroup		Case	Control	P	OR(95% CI)
Gender					
	Male	1371	1377	0.003	1.18(1.06–1.32)
	Female	512	596	0.001	1.38(1.15–1.65)
Age					
	<55	766	940	0.002	1.26(1.09–1.47)
	≥55	1117	1033	0.002	1.23(1.08–1.39)
Obesity					
	BMI ≤30	1708	1853	9.93×10−5	1.22(1.10–1.34)
	BMI >30	175	120	0.015	1.56(1.09–2.23)
Hypertension					
	Yes	1203	710	0.136	1.11(0.97–1.27)
	No	680	1263	1.49×10−5	1.35(1.18–1.55)
Diabetes					
	Yes	549	343	2.23×10−5	1.53(1.26–1.87)
	No	1334	1630	0.008	1.16(1.04–1.29)
Hyperlipidemia					
	Yes	938	980	0.001	1.24(1.09–1.41)
	No	945	993	0.003	1.22(1.07–1.38)
Smoking					
	Yes	971	815	0.005	1.22(1.06–1.40)
	No	912	1158	0.001	1.25(1.09–1.42)

% CI): odds ratio with 95% confidence interval. Obesity was defined as BMI >30. BMI: body mass index; P: P-value from logistic regression; OR (95

### NEXN promotes contractile phenotype transition of VSMCs and suppresses balloon-injury induced neointima formation

**Figure 1:**
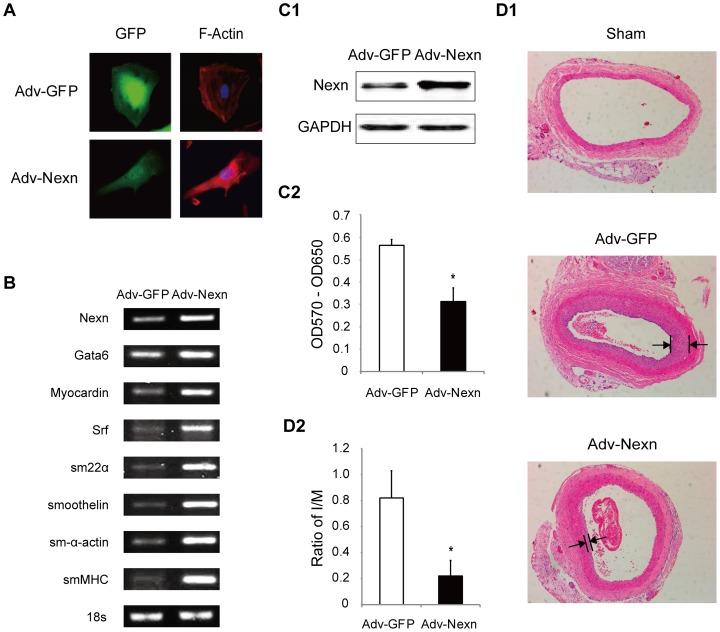
Nexn overexpression promotes the contractile phenotype of VSMCs and inhibits balloon injury-induced neointima formation. (A) VSMCs infected with Adv-Nexn with GFP reporter gene (green) showed a contractile phenotype with enhanced F-actin bundling (red) compared with Adv-GFP control (×200). Nuclei were stained in blue by DAPI. (B) Expression of contractile markers was induced in VSMCs infected with Adv-Nexn by RT-PCR, 18 s as loading control. (C) VSMCs was infected with Adv-Nexn, expression of Nexn was detected by western-blot (C1), and cell growth was arrested by Nexn with MTT assay. Data presented are mean ± SD from at least 3 independent experiments. *P<0.05 by Student's *t*-test. (D) Balloon injury was performed in the right common carotid artery with adenovirus containing Ad-GFP or Ad-Nexn infused into the injured arteries. 2 weeks after balloon injury, control uninjured and balloon-injured arteries were isolated, sectioned, stained with H & E (D1), and I/M ratios (intima/media) were calculated (D2). *P<0.05 by Student's *t*-test (*n* = 6 rats).

To find out the functional link of NEXN and CAD, we next explored the role of NEXN in vessels. Nexn is specifically expressed in rat VSMCs and downregulated by various inflammatory factors, including PDGF-BB, TNF-alpha and FGF2, which induced synthetic phenotype of VSMCs (Figure S1). These data suggest a role of Nexn in maintaining contractile phenotype of VSMCs. We then overexpressed Nexn in VSMCs by adenovirus infection. Fluorescence staining showed more enhanced F-actin bundles of VSMCs in Nexn group compared with control (Figure 1A). Assembly of actin cytoskeleton into elongated stress fibers is a hallmark of the VSMC contractile state. The mRNA level of SMC-specific contractile markers such as sm-α-actin, smoothelin, smMHC and SM22α was markedly increased by Adv-Nexn treatment (Figure 1B). Besides, transcriptional factors including GATA-6, serum response factor (SRF) and myocardin, which regulate VSMC marker genes, were activated as well. How NEXN regulates the transcription factors need to be explored in further studies. In parallel, cell proliferation was inhibited by Nexn (Figure 1C). Finally, we detected the effect of Nexn on *in vivo* arteries under a pathological remodeling induced by balloon injury. Adenovirus expressing either Nexn or GFP control (10^10^ pfu/ml) was infused into the injured right common carotid artery and 2 weeks after injury, arteries were isolated and morphometric analysis was performed. Nexn significantly inhibited neointima formation (Figure 1D). Collectively, these results indicate a causal role of Nexn in promoting VSMC phenotype transition into the contractile state, which provides functional evidence of NEXN involved in CAD pathogenesis.

### Risk allele T of rs1780050 is correlated with decreased expression of NEXN

**Figure 2:**
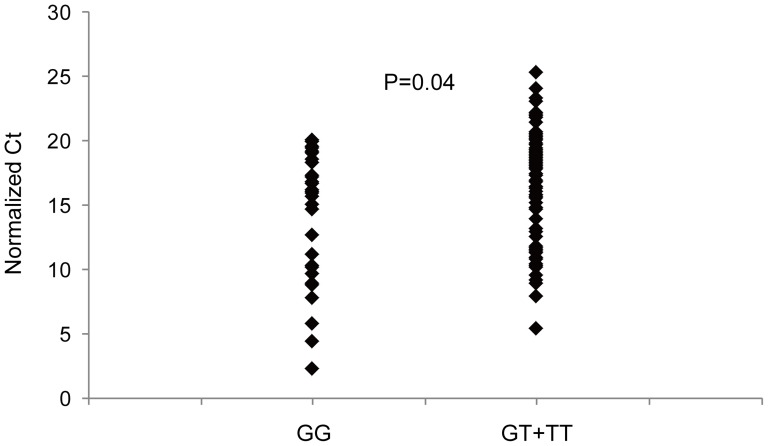
Risk allele T of rs1780050 correlates with reduced expression of NEXN. 121 healthy individuals were randomly recruited and genotyped by direct sequencing. The individuals were divided into GG and GT+TT group. The expression level of NEXN was quantified by real-time PCR and presented as normalized Ct, which was inversely correlated with the true amount of NEXN transcripts. The average normalized Ct is compared between GG group and GT+TT group by Student's t-test.

NEXN play a role in promotion of contractile phenotype of VSMCs, making it a novel candidate of CAD susceptibility genes. In order to test whether the associated SNP rs1780050 has a functional effect, we tested correlation between the SNP genotypes and NEXN expression using eQTL. 121 healthy individuals were genotyped by direct sequencing. Among them 34 individuals were genotyped as GG, and 87 individuals were GT+TT (60 were GT and 27 were TT, respectively). The relative expression of NEXN by real-time PCR was presented as normalized Ct, which was inversely correlated with the true amount of NEXN transcripts. The average normalized Ct of GG group was 14.6 and that of GT+TT group was 16.7, with a significant P value of 0.04 (Figure 2). We further compared the average normalized Ct between GT and TT groups, and no significant difference was found, which may be due to the inherent dominant mode of risk allele T over G allele. These results indicated the risk allele T decreased the expression of NEXN, thus contributing to a higher risk of CAD susceptibility in the population.

## Discussion

NEXN is a muscle-specific F-actin protein. In this study, we explored the association of genetic variants in *NEXN* with human vascular disease. We identified *NEXN* as a novel susceptibility gene for CAD, and then we provided the following functional evidence to support NEXN as a causal factor of CAD: 1) NEXN was downregulated in response to inflammatory cytokines in smooth muscle cells; 2) NEXN promotes VSMC to a contractile phenotype and showed protective effects in injured vessels; 3) Risk T allele of rs1780050 is associated with decreased level of NEXN by eQTL analysis. Taken together, our findings provide novel evidence that an F-actin regulator in VSMC contributes to CAD in human populations.

The actin cytoskeleton in VSMC is a highly dynamic structural network. Actin-dependent events have been implicated in cellular morphogenesis, proliferation, migration, and cell differentiation. It has been already established that actin stabilization is essential in maintaining proper function of VSMC. Altered ratio of F actin/G actin triggered signal transduces to the nucleus, activating the actin-MRTF-SRF gene regulatory axis, and responsive smooth muscle specific genes, leading to cell phenotype modulation [22], [23]. Mutations in smooth muscle alpha-actin (ACTA2) caused inappropriate proliferation of VSMCs, contributing to stenosis of arteries [24]. Our study provides novel evidence that F-actin binding protein facilitates contractile phenotype transition of VSMCs, and genetic variants of *NEXN* contribute to CAD in human populations.

In this study, we selected and genotyped five tagging SNPs in three independent CAD populations. The associated SNP rs1780050 could be validated in 3 independent populations, which strongly indicates *NEXN* as a CAD susceptibility gene. Given SNP rs1780050 is located in the intron of *NEXN*, whether rs1780050 is a functional variant *per se*, like acting as an enhancer to regulate NEXN expression, or in linkage disequilibrium with the true causal variant(s) is unknown. By eQTL analysis, we further demonstrated the risk allele T of SNP rs1780050 is correlated with reduced expression of NEXN in healthy individuals. Combined with the above functional implication of NEXN in contractile phenotype transition of VSMCs, our results strongly suggested that impaired expression of NEXN increases the susceptibility to CAD.

Of note, the genetic predisposition of rs1780050 to CAD exhibited several features in corporation with other risk factors. Firstly, rs1780050 was significantly associated with CAD in non-hypertensive group but not in hypertensive group. Meanwhile, there is no significant association between rs1780050 and hypertension in this study (Table S5). It is tempting to speculate that NEXN regulates VSMC function to participate in both hypertension and CAD. Secondly, gender, age, obesity, diabetes, hyperlipidemia and smoking do not significantly increase its susceptibility to CAD, suggesting NEXN acting as an independent factor to the pathogenesis of CAD.

Due to a limited number of SNPs genotyped in this study, the independence or structures of population 1 and 2 could not be evaluated by, for example, principal component analysis, which requires a large number of SNPs.

When these findings are interpreted, several study limitations must be taken into consideration. First, as a population based study, the association of *NEXN* with CAD in Han Chinese must be validated in other populations. Secondly, although we demonstrated the risk allele T of SNP rs1780050 is correlated with reduced expression of NEXN in healthy individuals by eQTL analysis, we still could not exclude the existence of other functional variants due to the limitation of eQTL method. Further functional studies on NEXN variants are needed.

In summary, our study demonstrated NEXN is a novel susceptibility gene for CAD, which provides evidence on the contribution of the F-actin regulator for VSMC phenotypic modulation and for coronary artery diseases.

## Supporting Information

Figure S1 **Nexn is downregulated by pro-inflammatory factors in VSMCs.** (A) Nexn expression in smooth muscle cells and aorta fibroblast cells isolated from rat aorta was detected by western-blot, GAPDH as an internal control. (B) Nexn expression in VSMC stimulated by inflammatory cytokines was detected by real-time PCR. Cultured cells were quiescent for 48 hours and treated with PDGF-BB (25 ng/ml), TGF-β (10 ng/ml) and FGF2 (25 ng/ml) for 8 hours.
(TIF)

Table S1 **Sequencing primers for 5 SNPs of** ***NEXN*** **.**
(DOC)

Table S2 **Sequence of MLPA probes for 5 SNPs of** ***NEXN*** **.**
(DOC)

Table S3 **PCR primers for cloning of rat** ***Nexn*** **.**
(DOC)

Table S4 **RT-PCR primers for target gene expression in VSMCs**.
(DOC)

Table S5 **Allelic association of rs1780050 with hypertension, diabetes and hyperlipidemia.**
(DOC)
