# Supplementary figures and images for: NEXN Is a Novel Susceptibility Gene for Coronary Artery Disease in Han Chinese
Source: PLoS One. 2013 Dec 11;8(12):e82135. doi: 10.1371/journal.pone.0082135 (PMC3859596; doi:10.1371/journal.pone.0082135)

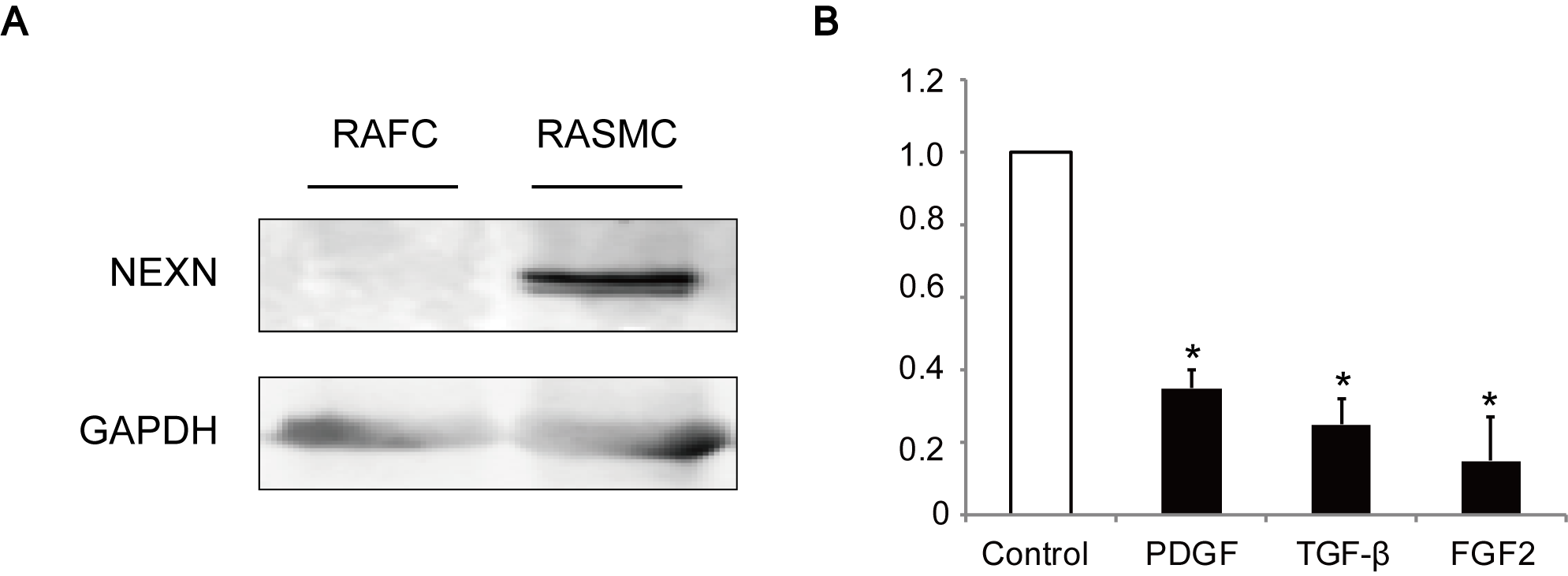

Supplement: Figure S1 — Nexn is downregulated by pro-inflammatory factors in VSMCs. (A) Nexn expression in smooth muscle cells and aorta fibroblast cells isolated from rat aorta was detected by western-blot, GAPDH as an internal control. (B) Nexn expression in VSMC stimulated by inflammatory cytokines was detected by real-time PCR. Cultured cells were quiescent for 48 hours and treated with PDGF-BB (25 ng/ml), TGF-β (10 ng/ml) and FGF2 (25 ng/ml) for 8 hours. (TIF) [file pone.0082135.s001.tif]
