# Supplementary material for: NEXN Is a Novel Susceptibility Gene for Coronary Artery Disease in Han Chinese
Source: PLoS One. 2013 Dec 11;8(12):e82135. doi: 10.1371/journal.pone.0082135 (PMC3859596; doi:10.1371/journal.pone.0082135)
Supplement: Table S1 — Sequencing primers for 5 SNPs of NEXN . (DOC) [file pone.0082135.s002.doc]

**Table S1**

| **SNP** | **Forward Primer** | **Reverse Primer** |
| --- | --- | --- |
| rs1166706 | CCAGAGATCCAGGTCTTCCTATT | CTGAGGCTTCAGGAGTTCTGTT |
| rs1780045 | CATCTCCCCTACTAGAATTTACTCTATT | CTCGAACACCTGAACTCAGGT |
| rs1166698 | GGCAGTTTAAGTTAGCAGCAT | CTTCAGCTTGCTTCTTTCGGT |
| rs1780050 | CCCACTGTATGATTTTCTTCATCT | GGCAATGCTATGGCATGTTT |
| rs17101082 | GAATGTCAGAGGATTTTAGCCAT | CTTCTCTAAATGCAGGGTCCA |
