# Supplementary material for: NEXN Is a Novel Susceptibility Gene for Coronary Artery Disease in Han Chinese
Source: PLoS One. 2013 Dec 11;8(12):e82135. doi: 10.1371/journal.pone.0082135 (PMC3859596; doi:10.1371/journal.pone.0082135)
Supplement: Table S2 — Sequence of MLPA probes for 5 SNPs of NEXN . (DOC) [file pone.0082135.s003.doc]

**Table S2**

| **SNP** | **SNP target probe 1** | **SNP target probe 2** | **Ligation probe** |
| --- | --- | --- | --- |
| **rs1166706** | TACGACTCACTATAGGTGCGGAAGATGG | TACGACTCACTATAGGTAGTGCGGAAGATGA | TCTTTCATTGAAGGACACATGGAAAATGGCCGCTTTTCT |
| **rs1780045** | TACGACTCACTATAGGTCCAGTGAGCAC | TACGACTCACTATAGGTGATCCAGTGAGCAT | ATATAATGTATTACTTACACCGCTTTTCTGGATTCATCG |
| **rs1166698** | TACGACTCACTATAGGTAATCACTTTCTCCCG | TACGACTCACTATAGGTAGTAATCACTTTCTCCCA | GAAAATTGAAACTAACTTTTGGCTTGCCGAATATCAT |
| **rs1780050** | TACGACTCACTATAGGTACTGGTTCCTAGGG | TACGACTCACTATAGGTCAGACTGGTTCCTAGGT | TATAAATTGGTGAGAGGACGGGTGTGGCGGACCGCTATC |
| **rs17101082** | TACGACTCACTATAGGTAATGGTGCTACAA | TACGACTCACTATAGGTCTGAATGGTGCTACAG | ATTTAAGGCATTATTGAAAGGACCGCTATCAGGACATAG |
