# Supplementary material for: NEXN Is a Novel Susceptibility Gene for Coronary Artery Disease in Han Chinese
Source: PLoS One. 2013 Dec 11;8(12):e82135. doi: 10.1371/journal.pone.0082135 (PMC3859596; doi:10.1371/journal.pone.0082135)
Supplement: Table S3 — PCR primers for cloning of rat Nexn . (DOC) [file pone.0082135.s004.doc]

**Table S3**

| **Primer name** | **Primer sequence (5'-3')** |
| --- | --- |
| rNexilin_F1 | CATGCTAGCATGAATGACGTGTCACAGAAG |
| rNexilin_R1 | GGCTTGAATTGAACCACTCCT |
| rNexilin_F2 | GAAACAGGAATTTGAACAACT |
