# Supplementary material for: NEXN Is a Novel Susceptibility Gene for Coronary Artery Disease in Han Chinese
Source: PLoS One. 2013 Dec 11;8(12):e82135. doi: 10.1371/journal.pone.0082135 (PMC3859596; doi:10.1371/journal.pone.0082135)
Supplement: Table S4 — RT-PCR primers for target gene expression in VSMCs. (DOC) [file pone.0082135.s005.doc]

**Table S4**

| **Gene** | **Forward primer (5'-3')** | **Reverse primer (5'-3')** |
| --- | --- | --- |
| rNEXN | ACAGTGTCTCAAGAATCTCT | GGCTTGAATTGAACCACTCCT |
| rGATA6 | CAACGCATGCGGTCTCTACA | ACAGTCCAAGCCGTCGTGAT |
| rMyocardin | CCCTTCCACCAAACCTGGAT | AGGGTTGCCAGCACAATCCT |
| rSRF | CGGAGCCTGAGCGAGAT | TGGCAAAGGTATACACAT |
| rSM22α | CATGGTCTTCAAGCAGAT | CATAAACCAGTTGGGATCT |
| rSmoothelin | CAACAGCATCAAGCAGAT | GCACCATGTCCTCTGTAT |
| rsm-α-actin | ACACGGCATCATCACCAACT | CGTTAGCAAGGTCGGATGCT |
| rsmMHC | GACATGCCTCAATGAGGCTT | GAGTAGATGGGTAGGTGCTT |
| 18S | CGAACGTCTGCCCTATCAACT | CCCTCCAATGGATCCTCGTT |
