# Supplementary material for: NEXN Is a Novel Susceptibility Gene for Coronary Artery Disease in Han Chinese
Source: PLoS One. 2013 Dec 11;8(12):e82135. doi: 10.1371/journal.pone.0082135 (PMC3859596; doi:10.1371/journal.pone.0082135)
Supplement: Table S5 — Allelic association of rs1780050 with hypertension, diabetes and hyperlipidemia. (DOC) [file pone.0082135.s006.doc]

**Table S5**

|  | **Case** | **Control** | **MAF(case/control)** | **P** | **OR(95% CI)** |
| --- | --- | --- | --- | --- | --- |
| Hypertension | 1913 | 1943 | 0.495/0.473 | 0.175 | 1.07(0.97-1.17) |
| Diabetes | 892 | 2964 | 0.484/0.484 | 0.521 | 0.97(0.87-1.08) |
| Hyperlipidemia | 1918 | 1938 | 0.488/0.480 | 0.613 | 1.02(0.94-1.12) |
